# Cutaneous adverse reactions specific to epidermal growth factor receptor inhibitors

**Published:** 2015

**Authors:** I Lupu, VM Voiculescu, N Bacalbasa, BE Prie, I Cojocaru, C Giurcaneanu

**Affiliations:** * “Carol Davila” University of Medicine and Pharmacy, Bucharest, Romania; ** Department of Dermatology, “Elias” University Hospital, Bucharest, Romania

**Keywords:** cutaneous adverse reactions, inhibitors of the EGF receptors, oncology

## Abstract

Classical antineoplastic therapy is encumbered by extensively studied adverse reactions, most often of systemic nature. The emergence of new generations of anticancer treatments, including epidermal growth factor receptor inhibitors, besides improving the response to treatment and the survival rate, is accompanied by the occurrence of new specific side effects, incompletely studied. These side effects are most often cutaneous (hand foot syndrome, acneiform reactions), and in some cases are extremely severe, requiring dose reduction or drug discontinuation. The prevention of the cutaneous adverse effects and their treatment require a close collaboration between the oncologist and the dermatologist. The occurrence of some of these skin adverse effects may be a favorable prognostic factor for the response to the cancer treatment and the overall survival.

**Abbreviations:** EGFR = epidermal growth factor receptors; EGFRI = epidermal growth factor receptors inhibitors

## Introduction

New chemotherapeutic agents have the ability to specifically target cancer cells. They assure an increased survival and less systemic toxicities, compared to conventional cytotoxic chemotherapies [**1**,**2**]. Despite this, targeted chemotherapies have numerous cutaneous adverse reactions, which may cause serious discomfort and negatively affect compliance to treatment. The presence and severity of cutaneous adverse event have a positive correlation with the patient’s response to treatment and overall survival, especially for epidermal growth factor receptor inhibitors [**3**].

## Epidermal Growth Factor Receptor Inhibitors

EGFR is a transmembrane tyrosine kinase receptor, whose overexpression causes gene amplification and mutation, leading to cell proliferation, cell survival, ability of invasion and metastasis, tumor-induced neoangiogenesis [**4**]. EGFR inhibitors are targeted chemotherapy agents approved for the treatment of many advance-stage epithelial cancers (non-small cell lung cancer, colorectal cancer, squamous cell carcinoma of the head and neck) [**4**,**5**]. There are two classes of EGFR inhibitors: **monoclonal antibodies** (cetuximab, panitumumab, matuzumab) that bind to the extracellular tyrosine kinase domain of EGFR; and **small-molecule tyrosine kinase inhibitors** (gefitinib, erlotinib, lapatinib, afatinib) which target the intracellular domain [**4**]. EGFR inhibitors target aberrantly activated or overexpressed EGFR in tumor cells, causing cellular apoptosis by inhibiting metastasis, growth, proliferation, differentiation and angiogenesis [**6**]. EGFR inhibitors have a good safety profile compared with classical cytotoxic chemotherapies. They cause frequent cutaneous adverse events because EGFR is highly expressed in the skin and adnexal structures (mainly in the basal and suprabasal keratinocytes, the outer root sheath of hair follicles, sebaceous epithelium) [**7**]. The papulopustular rash and xerosis are the most common cutaneous adverse reactions. Less frequent, patients develop paronychia, abnormal scalp, facial hair, and/ or eyelash growth, maculopapular rash, mucositis and post inflammatory hyperpigmentation [**7**]. These adverse events can impair the patient’s quality of life and adherence to treatment and in severe cases may require dose reduction or even temporary/ permanent interruption of therapy [**8**].

## Papulopustular rash

Papulopustular rash is the most common cutaneous adverse effect of EGFRI, which occurs in 80% of the patients early in the course of treatment [**7**,**9**]. Although terms like acneiform, acne-like and even acne have been used to describe this rash, it differs from acne from the clinical, histopathological and therapeutical point of view. The rash manifests itself by folliculocentric erythematous papules and pustules that predominately affect seborrheic-rich areas (scalp, face- particularly the nose, nasolabial folds, perioral region, upper trunk and “V” region of the neck and chest) [**14**]. The periorbital region and the palmoplantar surfaces are usually spares [**16**]. Unlike acne, there are no comedones, lesions can extend to the lower trunk, extremities and buttocks and can be associated with pruritus, pain, stinging, irritation [**7**,**15**]. The onset typically occurs in the first two weeks of treatment, but it can vary from as early as 2 days to as late as 6 weeks [**7**].

The rash evolves through four stages [**22**]

- First week: dysesthesia with erythema and edema

- Second and third week: eruption of papulopustular lesions

- Third and fourth week: crusts formation

- One month and longer: persistent erythema, xerosis and telangiectasia in the area affected by the rash

The evolution of the rash is characterized by waxing and waning of lesions. The vast majority of patients present partial or complete resolution of the lesions despite continuing the treatment with EGFI. Complete resolution can be seen 4 weeks after treatment discontinuation [**23**,**24**].

The rash may cause long-term cutaneous sequelae like post-inflammatory hyperpigmentation, telangiectasia and erythema [**25**].

EGFR are expressed in the undifferentiated basal and suprabasal keratinocytes, outer layer of the hair follicles and the sebaceous glands, with a critical role in regulating keratinocyte proliferation, differentiation, migration and survival [**18**]. The inhibition of this receptor results in reduced proliferation, diminished growth and apoptosis of keratinocytes, decreased cell migration and enhanced differentiation [**19**]. The inflammatory response mediated by keratinocyte-derived cytokines, recruits macrophages, mast cells and granulocytes [**20**]. This inflammatory response can be caused by the alteration of the growth and differentiation of follicular epithelium, which results in follicular occlusion, rupture of follicular infundibulum and neutrophils chemotaxis [**21**]. The initial lesion is a sterile neutrophilic suppurative infiltrate. Due to a compromised epidermal barrier, infection with Staphylococcus aureus can play a role in the late phases of the eruption, but the initial folliculitis is sterile [**21**].

EGFR inhibitors and radiotherapy have a synergistic anti-tumor activity, but radiotherapy increases EGFRI side effects [**44**]. Papulopustular eruptions are dose dependent and those associated with monoclonal antibodies tend to be more frequent and severe compared with low-molecular tyrosine kinase inhibitors [**17**]. Papulopustular rash could be a surrogate marker for efficacy of EGFRI treatment [**10**-**12**]. Patients who developed a papulopustular rash while on erlotinib or gefitinib for non-small cell lung cancer had a lower risk of death (HR 0.30; 95% CI 0.21–0.43) and disease progression (HR 0.50; 95% CI 0.41–0.61) than patients without a rash [**12**]. Patients who developed more than one type of cutaneous toxicity proved to have an even better survival rate [**26**]. Increasing the dose for patients with grade ≤1 may improve the response rate to treatment [**13**].

As for all cutaneous adverse reactions, the severity of papulopustular rash due to EGFRI are grated according to the National Cancer Institute Common Terminology for Adverse Events (CTCAE) version 4.0 (see Table 1) [**27**]: Grade 1: papules and/ or pustules covering <10% of the body-surface area (BSA) with or without symptoms of pruritus or tenderness, Grade 2: papules and/ or pustules covering 10–30% of the BSA with or without symptoms of pruritus or tenderness; with psychosocial impact, Grade 3: papules and/ or pustules covering >30% of the BSA with or without symptoms of pruritus or tenderness; limiting self-care activities of daily living, associated with local superinfection with oral antibiotics indicated, Grade 4: covering any percentage of the BSA with or without 4 Dermatology Research and Practice symptoms of pruritus or tenderness; associated with extensive super-infection with intravenous antibiotics indicate life threatening consequences, and Grade 5: Death Therapy can be divided into prophylactic and active treatment [**35**].

General measures include avoiding sun exposure, hot showers, lotions that contain alcohols, perfumed soaps and detergents and the use of broad-spectrum sunscreen with SPF >30 and moisturizing creams twice a day [**35**].

Prophylactic treatment with oral tetracyclines (doxycycline or minocycline each 100mg twice a day) together with low-potency topical corticosteroids twice a day on face and chest for 6 to eight weeks can be started in the same manner as the EGFRI therapy [**36**].

The severity of the eruptions guides the approach to treatment [**30**-**33**,**36**]:

Patients with grade 1 eruption can receive low potency topical corticosteroids and topical antibiotics (clindamycin gel 1%; erythromycin gel/ cream 3%; metronidazole cream/ gel 0.75%-1%) twice a day for 4 weeks. Patients are reevaluated after 2 weeks, and if the eruption does not improve, they are treated as those with grade 2 eruption.

Patients with grade 2 rash require low potency topical corticosteroids twice a day on the face and neck and fluocinonide cream 0,05% twice a day on chest and back, in association with tetracycline (doxycycline 200mg per day or minocycline 200 mg per day) for 4 to 6 weeks. Doxycycline is safer especially in patients with renal impairment and minocycline is less photosensitizing.

If the patient was already on an oral tetracycline, he can receive a first generation oral cephalosporin (cephalexin 500mg twice per day, cefadroxil 500mg twice per day) or trimetroprim 160mg/ sulfamethoxazole 800mg twice per day for 4 weeks.

Patients are reevaluated after 2 weeks of treatment and if no improvement can be found, they should be treated as those with a grade ≥ 3 eruption.

Patients with grade ≥ 3 eruption, intolerable grade 2 rash, rash that interferes with self-care activities – adjusting the dose of EGFR inhibitors until the rash improves to grade ≤ 2; accompanied by oral antibiotics such as doxycycline 100mg twice per day or minocycline 100mg twice per day, first generation oral cephalosporin (cephalexin 500mg twice per day; cefadroxil 500mg twice per day) or trimetroprim 160mg/ sulfamethoxazole 800mg twice per day for 4 weeks in association with oral prednisone 0,5 mg/ kg up to a maximum of 40mg/ day for 7 days. Patients are reevaluated after 2 weeks of treatment.

Refractory grade ≥ 3 rash oral tetracycline are discontinued before starting low dose isotretinoin (20-30mg per day) for at least two months [**30**]. A significant improvement can be seen after 4 weeks [**34**]. Isotretinoin may cause or aggravate xerosis, photosensitivity and cheilitis. Emollients and photoprotection may improve some of these symptoms.

In case none of these measures show any improvement, dose adjustment, interruption or discontinuation of EGFRI could be taken into consideration.

Pruritus can be alleviated with oral H1antihistamines sedative or non-sedative [**28**], aprepitant (a neurokinin 1 receptor antagonist); GABA agonists (gabapentin, pregabalin) can be used in case oral antihistamines are ineffective [**29**]. Pruritus can also occur as a result of xerosis and general measures to prevent dryness should be employed.

## Xerosis and fissures

Xerosis is the second most common cutaneous adverse reaction to EGFRI that affects up to 35% of the patients [**38**]. It presents itself as dry, itchy, scaly patches mostly limited to regions affected by papulopustular rash, but tending to involve a larger surfer area [**17**]. It can evolve with fissures of tips of fingers, toes, dorsal aspect of interphalangeal joints, and asteatotic eczema. Older people with personal history of eczema and prior exposure to cytotoxic therapy are more frequently affected [**7**]. Treatment requires topical emollients, short-term low-potency topical corticoids in case of asteatotic eczema. Disruption of the normal skin barrier can lead to superinfection with Staphylococcus aureus and rarely herpes simplex virus [**37**]. In case superinfection is suspected, cultures should be obtained and treatment with oral or topical antibiotics instituted [**37**]. Fissures can be treated with hydrocolloid dressings, occlusive propylene glycol solutions or liquid cyanoacrylate glue [**7**].

## Hair changes

A variety of hair changes have been described in patients treated with EGFRI, such as trichomegaly (elongation and curling of eyelashes), hypertrichosis and scalp changes (brittleness, slow growth, scarring/ non-scarring alopecia) [**7**]. Hirsutism and trichomegaly are a rare side effect that develops after 2-5 months of treatment with EGFRI. They persist for the entire duration of the treatment. Eyelashes become wavy, curly and aberrant, and may cause corneal irritation and even ulceration [**7**]. They can be clipped at every 2-4 weeks, and patient who still experiences irritation can be referred to the ophthalmologist. Hirsutism can be treated with laser hair removal with/ without topical eflornithine cream [**41**].

Non-scarring alopecia appears after 2-3 months of treatment in the form of frontal or patchy patterns. It may progress to diffuse alopecia, and resolves after discontinuation of therapy, although the hair quality may not be the same. Minoxidil 2%, 5% can be employed in the treatment of non-scaring alopecia. Follicular pustules can cause scarring alopecia of the scalp, facial and chest hair. Prevention and treatment of papulopustular rash should be employed in order to prevent scaring alopecia. Treatment options include topical hydrocortisone 0.2%, steroid shampoos and class I steroid lotions [**39**], bath oils or mild shampoo followed by antibiotic spray [**40**].

## Nail changes

Nail changes appear after two or more months of treatment, typically in the form of paronychia (painful inflammation of the nail fold) and periungual pyogenic granuloma-like lesions [**7**]. Nail matrix inflammation can cause subsequent onycholysis and onychodystrophy. Paronychia is characterized by tender inflammation of the nail fold of finger and toes, due to the direct inhibition of keratinocytes in the nail matrix [**7**]. The first fingers and toes are most frequently affected. Paronychia is not an infectious process but has the potential to become superinfected [**7**]. Cultures may be necessary to determine the proper antibiotic treatment. Treatment is aimed at reducing periungual trauma and inflammation, preventing superinfections and eliminating excessive granulation formation. Topical corticosteroid, topical calcineurin inhibitors and oral tetracycline can reduce periungual inflammation [**42**]. Excessive granulation formation can be treated by electrocauterisation, silver nitrate and nail avulsion [**43**].

## Maculopapular rash

Maculopapular appears later than the papulopustular rash especially on the face and limbs. It is more pruritic, can become chronic and is accompanied by a dry pulpitis of the fingers, feet xerosis and photosensitivity. The treatment requires topical corticosteroids, antihistamines and sunscreen.

## Oral complications

EGFRI can cause mild to moderate mucositis, stomatitis, aphthous-like ulcers, xerosis of vaginal and perineal mucosa, that appear in the course of treatment and usual resolve themselves without specific intervention [**22**]. EGFRI can cause severe mucositis in combination with radiotherapy or cytotoxic agents [**7**].Oral hygiene and a soft diet may prevent oral mucositis. Local anesthetics and antiseptics, mucosal-coating agents (magic mouthwash) and systemic analgesics can be used. Dose modification or treatment interruption can be necessary in case of severe mucositis.

## Conclusion

Cutaneous adverse reactions are common side effects of epidermal growth therapy having a significant impact on the patient’s physical, emotional and psychosocial health and may affect compliance to treatment. Severe impairment requires dose reduction or temporary or definitive interruption therapy, preventing the complete administration of treatment at an effective dose and for an optimal period. Therefore, guidelines for prevention and management of these the skin toxicities of antineoplastic therapy must be established and implemented.

**Disclosure**

None

**Acknowledgement**

This paper is supported by the Sectorial Operational Programme Human Resources Development (SOP HRD), financed from the European Social Fund and by Romanian Government under the contract number POSDRU/159/1.5/S/137390.

## References

[1] Ricciardi S, Tomao S, de Marinis F (2009). Toxicity of targeted therapy in non-small-cell lung cancer management. Clin Lung Cancer.

[2] Vokes EE, Chu E (2006). Anti-EGFR therapies: clinical experience in colorectal, lung, and head and neck cancers. Oncology.

[3] Liu HB, Wu Y, Lv TF (2013). Skin rash could predict the response to EGFR tyrosine kinase inhibitor and the prognosis for patients with non-small cell lung cancer: a systematic review and meta-analysis. PLoS ONE.

[4] Ciardiello F, Tortora G (2008). EGFR antagonists in cancer treatment. The New England Journal of Medicine.

[5] Mendelsohn J, Baselga J (2006). Epidermal growth factor receptor targeting in cancer. Semin Oncol.

[6] Harari PM, Allen GW, Bonner JA (2007). Biology of interactions: anti-epidermal growth factor receptor agents. Journal of Clinical Oncology.

[7] Hu JC, Sadeghi P, Pinter-Brown LC (2007). Cutaneous side effects of epidermal growth factor receptor inhibitors: clinical presentation, pathogenesis, and management. J Am Acad Dermatol.

[8] Boone SL, Rademaker A, Liu D (2007). Impact and management of skin toxicity associated with anti-epidermal growth factor receptor therapy: survey results. Oncology.

[9] Pérez-Soler R, Delord JP, Halpern A (2005). HER1/ EGFR inhibitor-associated rash: future directions for management and investigation outcomes from the HER1/ EGFR inhibitor rash management forum. Oncologist.

[10] Perez-Soler R (2006). Rash as a surrogate marker for efficacy of epidermal growth factor receptor inhibitors in lung cancer. Clin Lung Cancer.

[11] Wacker B, Nagrani T, Weinberg J (2007). Correlation between development of rash and efficacy in patients treated with the epidermal growth factor receptor tyrosine kinase inhibitor erlotinib in two large phase III studies. Clin Cancer Res.

[12] Petrelli F, Borgonovo K, Cabiddu M (2012). Relationship between skin rash and outcome in non-small-cell lung cancer patients treated with anti-EGFR tyrosine kinase inhibitors: a literature- based meta-analysis of 24 trials. Lung Cancer.

[13] Van Cutsem E, Tejpar S, Vanbeckevoort D (2012). Intrapatient cetuximab dose escalation in metastatic colorectal cancer according to the grade of early skin reactions: the randomized EVEREST study. J Clin Oncol.

[14] Roé E, García Muret MP, Marcuello E (2006). Description and management of cutaneous side effects during cetuximab or erlotinib treatments: a prospective study of 30 patients. J Am Acad Dermatol.

[15] Joshi SS, Ortiz S, Witherspoon JN (2010). Effects of epidermal growth factor receptor inhibitor-induced dermatologic toxicities on quality of life. Cancer.

[16] Belloni B, Schonewolf N, Rozati S, Goldinger SM, Dummer R (2012). Cutaneous drug eruptions associated with the use of new oncological drugs. Chemical Immunology and Allergy.

[17] Li T, Perez-Soler R (2009). Skin toxicities associated with epidermal growth factor receptor inhibitors. Target Oncol.

[18] Mendelsohn J, Baselga J (2003). Status of epidermal growth factor receptor antagonists in the biology and treatment of cancer. J Clin Oncol.

[19] Lacouture ME (2006). Mechanisms of cutaneous toxicities to EGFR inhibitors. Nat Rev Cancer.

[20] Lichtenberger BM, Gerber PA, Holcmann M (2013). Epidermal EGFR controls cutaneous host defense and prevents inflammation. Sci Transl Med.

[21] Busam KJ, Capodieci P, Motzer R (2001). Cutaneous side-effects in cancer patients treated with the antiepidermal growth factor receptor antibody C225. Br J Dermatol.

[22] Lynch TJ Jr., Kim ES, Eaby B, Garey J, West DP, Lacouture ME (2007). Epidermal growth factor receptor inhibitor-associated cutaneous toxicities: an evolving paradigm in clinical management. Oncologist.

[23] Hidalgo M, Siu LL, Nemunaitis J (2001). Phase I and pharmacologic study of OSI-774, an epidermal growth factor receptor tyrosine kinase inhibitor, in patients with advanced solid malignancies. J Clin Oncol.

[24] Li T, Perez-Soler R (2009). Skin toxicities associated with epidermal growth factor receptor inhibitors. Target Oncol.

[25] Burtness B, Anadkat M, Basti S (2009). NCCN Task Force Report: Management of dermatologic and other toxicities associated with EGFR inhibition in patients with cancer. J Natl Compr Canc Netw.

[26] Chanprapaph K, Pongcharoen P, Vachiramon V Cutaneous adverse events of epidermal growth factor receptor inhibitors: a review of 99 cases.

[27] Chen AP, Setser A, Anadkat MJ (2012). Grading dermatologic adverse events of cancer treatments: the Common Terminology Criteria for Adverse Events Version 4.0. J Am Acad Dermatol.

[28] Potthoff K, Hofheinz R, Hassel JC (2011). Interdisciplinary management of EGFR-inhibitor-induced skin reactions: a German expert opinion. Ann Oncol.

[29] Porzio G, Aielli F, Verna L (2006). Efficacy of pregabalin in the management of cetuximab- related itch. J Pain Symptom Manage.

[30] Lacouture ME, Anadkat MJ, Bensadoun RJ (2011). Clinical practice guidelines for the prevention and treatment of EGFR inhibitor-associated dermatologic toxicities. Support Care Cancer.

[31] Thatcher N, Nicolson M, Groves RW Thatcher N, Nicolson M, Groves RW et al. Expert consensus on the management of erlotinib-associated cutaneous toxicity in the U.K. Oncologist.

[32] Balagula Y, Lacouture ME, Cotliar JA (2010). Dermatologic Toxicities of Targeted Anticancer Therapies. Journal of Supportive Oncology.

[33] (2014). Epidermal Growth Factor Receptor Inhibitors: A Review of Cutaneous Adverse Events and Management Dermatology Research and Practice.

[34] Requena C, Llombart B, Sanmartín O (2012). Acneiform eruptions induced by epidermal growth factor receptor inhibitors: treatment with oral isotretinoin. Cutis.

[35] Balagula Y, Garbe C, Myskowski PL (2011). Clinical presentation and management of dermatological toxicities of epidermal growth factor receptor inhibitors. Int J Dermatol.

[36] Lacouture ME, Anadkat MJ, Bensadoun RJ (2011). Clinical practice guidelines for the prevention and treatment of EGFR inhibitor-associated dermatologic toxicities. Support Care Center.

[37] Segaert S, Van Cutsem E (2005). Clinical signs, pathophysiology and management of skin toxicity during therapy with epidermal growth factor receptor inhibitors. Ann Oncol.

[38] Lynch TJ Jr., Kim ES, Eaby B, Garey J, West DP, Lacouture ME (2007). Epidermal growth factor receptor inhibitor-associated cutaneous toxicities: an evolving paradigm in clinical management. Oncologist.

[39] Burtness B, Anadkat M, Basti S (2009). NCCN Task Force Report: management of dermatologic and other toxicities associated with EGFR inhibition in patients with cancer. J Natl Compr Canc.

[40] Ocvirk J, Cencelj S (2010). Management of cutaneous side-effects of cetuximab therapy in patients with metastatic colorectal cancer. JEADV.

[41] Hamzavi I, Tan E, Shapiro J, Lui H (2007). A randomized bilateral vehicle-controlled study of eflornithine cream combined with laser treatment versus laser treatment alone for facial hirsutism in women. J Am Acad Dermatol.

[42] Tosti A, Piraccini BM, Ghetti E, Colombo MD (2002). Topical steroids versus systemic antifungals in the treatment of chronic paronychia: an open, randomized double-blind and double dummy study. J Am Acad Dermatol.

[43] Ghodsi SZ, Raziei M, Taheri A, Karami M, Mansoori P, Farnaghi F (2006). Comparison of cryotherapy and curettage for the treatment of pyogenic granuloma: a randomized trial. Br J Dermatol.

[44] Bonner JA, Harari PM, Giralt J (2006). Radiotherapy pluscetuximab for squamous-cell carcinoma of the head and neck. The New England Journal of Medicine.

